# Microstructure—Machinability correlation in heat treated AISI 1040 steel: Comparative analysis of tool life and surface roughness

**DOI:** 10.1371/journal.pone.0352093

**Published:** 2026-06-22

**Authors:** Umanath R. Poojary, Ananda Hegde, Sriharsha Hegde, Rayappa Shrinivas Mahale

**Affiliations:** 1 Manipal Institute of Technology, Manipal Academy of Higher Education, Manipal, Karnataka, India; 2 Department of Automation and Robotics, JSPM’s Rajarshi Shahu College of Engineering, Pune, Maharashtra, India; King Mongkut's University of Technology North Bangkok, THAILAND

## Abstract

This study presents an integrated investigation of the machinability of heat treated AISI 1040 steel under annealed, normalized, and oil quenched conditions. The effects of heat treatment parameters, namely austenitizing temperature and soaking time, together with machining parameters including cutting speed, feed rate, and depth of cut, were evaluated in terms of tool life and surface roughness. Experiments were designed using a Taguchi L27 orthogonal array, and the responses were analyzed through analysis of variance (ANOVA), regression modeling, and optimization techniques. The results showed that temperature, cutting speed, and feed rate significantly influenced tool life, whereas feed rate was the most dominant factor affecting surface roughness in all heat treatment conditions. Annealed AISI 1040 steel exhibited the highest tool life and the best overall machinability because of its relatively softer ferrite-pearlite microstructure, while oil quenched specimens showed the lowest tool life due to the formation of hard martensitic phases. Normalized specimens displayed intermediate behavior, indicating a balanced combination of strength and machinability. The developed regression models showed good predictive capability for both tool life and surface roughness within the investigated parameter range. SEM analysis further confirmed that the observed variations in machinability were closely associated with microstructural evolution caused by different heat treatment routes. The study provides useful guidance for selecting suitable heat treatment and machining parameters to improve productivity, tool performance, and surface quality during machining of AISI 1040 steel.

## 1. Introduction

Medium carbon steels such as AISI 1040 are widely used in engineering applications including automotive shafts, gears, axles, and structural components due to their favorable combination of strength, toughness, and cost effectiveness. The mechanical and functional properties of these steels are significantly influenced by heat treatment processes, which alter the microstructure through controlled phase transformations. Common heat treatment methods such as annealing, normalizing, and quenching are employed to tailor properties like hardness, strength, and wear resistance based on specific application requirements [1–5].

Machinability is a critical aspect in manufacturing, especially for components requiring high dimensional accuracy and surface integrity. It is generally evaluated using parameters such as tool life and surface roughness, which directly affect production efficiency, tooling cost, and product quality. However, there exists an inherent trade off between mechanical strength and machinability. While heat treatment enhances strength and hardness, it often leads to reduced machinability due to increased cutting forces, higher temperatures at the tool,workpiece interface, and accelerated tool wear [6–7].

Previous studies have shown that machining parameters such as cutting speed, feed rate, and depth of cut play a significant role in determining tool wear and surface finish. Among these, cutting speed predominantly affects tool life due to thermal and diffusion wear mechanisms, whereas feed rate has been widely reported as the most influential parameter governing surface roughness. Additionally, the influence of heat treatment on machinability has been explored, where annealed steels with ferrite pearlite structures exhibit better machinability, normalized steels show moderate behavior due to refined grains, and quenched steels containing martensite present poor machinability owing to increased hardness and abrasiveness.

Dhar et al. [8] have reported about the possible benefit of providing the lubrication during the machining AISI 1040 steel. It is reported that the use of near dry lubrication leads to lower cutting temperature and cutting force. Shayea et al. [9] reported about achieving the reduced surface roughness by minimizing the vibration during the turning operation of AISI 1040 steel. Tiwari et al. [10] reported about the improvement of the machining performance of AISI 1040 steel by utilizing coconut oil as a cutting fluid with minimum quantity lubrication and cooling. Mudda et al. [11] reported about the comparison of various heat treatments which affect the properties of AISI 1040 steel which in turn affects the machinability of the material. Recent studies have also emphasized the role of sustainable machining approaches, including vegetable-oil-based cutting fluids, in reducing cutting temperature, tool wear, and environmental impact during machining operations. In addition, multi-attribute decision-making and hybrid optimization approaches have been increasingly used to identify suitable machining parameter combinations when multiple responses such as tool life, surface roughness, productivity, and quality are considered simultaneously [12].

Although several studies have investigated the effects of machining parameters and heat treatment independently, comprehensive studies combining both aspects along with statistical modeling and microstructural analysis remain limited for AISI 1040 steel. There is a lack of integrated investigations that simultaneously consider heat treatment parameters (temperature and soaking time), machining variables, and their interaction effects using advanced statistical tools such as ANOVA, regression modeling, and optimization techniques. Furthermore, limited work has been reported linking SEM based microstructural characterization directly with machinability responses such as tool life and surface roughness.

Although Taguchi-based machinability studies on AISI 1040 steel have been reported previously, most of them have primarily focused on the direct influence of machining parameters such as cutting speed, feed rate, and depth of cut on tool wear, cutting temperature, vibration, or surface roughness. Similarly, heat treatment studies on AISI 1040 steel have generally concentrated on hardness, impact toughness, or microstructural evolution rather than their combined influence on machinability. In contrast, the present study simultaneously considers heat treatment route, austenitizing temperature, soaking time, and machining parameters to establish a microstructure–machinability correlation under annealed, normalized, and oil-quenched conditions. Therefore, the novelty of this work lies not merely in the application of Taguchi–ANOVA analysis, but in combining statistical evaluation, regression-based prediction, optimization, hardness variation, and SEM-supported microstructural interpretation to explain the machining response of heat-treated AISI 1040 steel.

Therefore, the present study aims to establish a microstructure–machinability correlation for AISI 1040 steel subjected to annealing, normalizing, and oil quenching. The combined effects of austenitizing temperature, soaking time, cutting speed, feed rate, and depth of cut are evaluated using a Taguchi L27 experimental design. Tool life and surface roughness are considered as the machinability responses, while hardness and SEM observations are used to explain the role of microstructural changes. ANOVA is used to identify statistically significant parameters, regression models are developed for response prediction, and optimization is performed to determine suitable process conditions. The study therefore provides an integrated framework for selecting heat treatment and machining parameters for improved machinability of AISI 1040 steel.

## 2. Materials and methods

### 2.1. Material

The material used in this investigation is AISI 1040 medium carbon steel, supplied in the form of hot rolled round bars. This grade is widely used in engineering applications due to its good balance of strength, ductility, and machinability. The chemical composition of the material as obtained by spectroscopy analysis is presented in Table 1.

**Table 1:** Chemical composition of AISI 1040 steel (wt.%).

C	Mn	Si	P	S	Fe
0.426	0.231	0.872	0.0017	0.0026	Balance

### 2.2. Heat treatment procedures

Heat treatment was carried out to obtain three distinct microstructural conditions, namely annealed, normalized, and oil quenched states. Specimens were heated in a muffle furnace at three different austenitizing temperatures (900°C, 925°C, and 950°C) and three soaking times (1 h, 1.5 h, and 2 h), as shown in Table 2. The values are selected based on the previous studies reported regarding the properties of the AISI 1040 steel [2].

**Table 2:** Heat treatment parameters.

Temperature (°C)	Time (h)
900	1
925	1.5
950	2

After soaking, different cooling methods were adopted to achieve the required microstructures:

Annealing: Furnace cooling to obtain coarse ferrite–pearlite structure.
Normalizing: Air cooling to produce refined and uniform grain structure.
Oil quenching: Rapid cooling using SAE 40 oil to obtain martensitic structure.

SAE 40 oil was selected as the quenching medium due to its moderate cooling rate, which helps in minimizing distortion and cracking in medium carbon steels.

Hardness measurements were carried out using standard Vickers microhardness testing methods, and average values were recorded for each condition.

### 2.3. Microstructural characterization

Microstructural analysis was performed using scanning electron microscopy (SEM) to examine the phase constituents and grain morphology. The specimens were prepared using standard metallographic procedures, including grinding, polishing, and etching with 2% nital solution. SEM images were used to identify ferrite, pearlite, and martensite phases and to correlate microstructural features with machinability characteristics.

### 2.4. Machinability tests

Machining experiments were conducted on a CNC turning center under dry cutting conditions. A carbide insert VNMG12t304 with carbide grade of WK20CT (HC K20) was used as the cutting tool for all experiments.

Machinability was evaluated using the following criteria:

Tool life is determined based on average flank wear (VB = 0.3 mm) as per ISO 3685 [13].
Surface roughness is measured using a surface profilometer in accordance with ISO 4287 [14].

The machining parameters selected for the study were based on previous literature and industrial practice. The levels of parameters considered are as follows:

Cutting speed: 80, 120, 160 m/min
Feed rate: 0.08, 0.16, 0.24 mm/rev
Depth of cut: 0.3, 0.6, 0.9 mm

### 2.5. Design of experiments and statistical analysis

The experiments were designed using a Taguchi L27 orthogonal array to systematically study the influence of five factors: temperature, soaking time, cutting speed, feed rate, and depth of cut, each at three levels.

The Taguchi L27 orthogonal array was selected because it enables the systematic evaluation of five factors at three levels with a reduced number of experiments compared with a full factorial design. The main objective of the present design was to identify the dominant main effects of heat treatment and machining parameters on tool life and surface roughness.

Statistical analysis was performed using Minitab software. Analysis of variance (ANOVA) was employed to determine the significance and contribution of each parameter on tool life and surface roughness. Regression modeling was carried out to develop predictive equations for the response variables. In addition, response surface methodology (RSM) was used to identify optimal combinations of process parameters for maximizing tool life and minimizing surface roughness.

## 3. Results and discussion

### 3.1. Hardness results for annealed condition

The Vickers microhardness details with different temperatures and time combinations are provided in the Table 3 below.

**Table 3:** Hardness results for Annealed AISI 1040 steel.

Temperature (°C)	Time (h)	Hardness (HV)
900	1	203
900	1.5	200
900	2	197
925	1	195
925	1.5	192
925	2	190
950	1	190
950	1.5	188
950	2	188

### 3.2. Machinability results for annealed condition

The experimental results for tool life and surface roughness obtained under annealed conditions are presented in Table 4. The results indicate a clear dependence of machinability on both heat treatment and machining parameters.

**Table 4:** Tool Life and Surface Roughness results for annealed AISI 1040 steel.

Temperature (°C)	Time (h)	Speed (m/min)	Feed(mm/rev)	DoC (mm)	Tool Life (min)	Surface Roughness (µm)
900	1.0	80	0.08	0.3	15.5	1.268
900	1.0	80	0.16	0.6	14.9	1.436
900	1.0	80	0.24	0.9	14.5	1.592
900	1.5	120	0.08	0.3	14.6	1.041
900	1.5	120	0.16	0.6	14.0	1.214
900	1.5	120	0.24	0.9	13.5	1.366
900	2.0	160	0.08	0.3	13.5	1.109
900	2.0	160	0.16	0.6	12.9	1.327
900	2.0	160	0.24	0.9	12.4	1.472
925	1.0	120	0.08	0.6	16.8	0.913
925	1.0	120	0.16	0.9	16.2	1.082
925	1.0	120	0.24	0.3	15.8	1.251
925	1.5	160	0.08	0.6	15.9	0.987
925	1.5	160	0.16	0.9	15.3	1.203
925	1.5	160	0.24	0.3	14.8	1.348
925	2.0	80	0.08	0.6	18.1	1.173
925	2.0	80	0.16	0.9	17.5	1.362
925	2.0	80	0.24	0.3	17.1	1.507
950	1.0	160	0.08	0.9	17.0	1.224
950	1.0	160	0.16	0.3	16.4	1.401
950	1.0	160	0.24	0.6	16.0	1.548
950	1.5	80	0.08	0.9	19.2	1.356
950	1.5	80	0.16	0.3	18.6	1.584
950	1.5	80	0.24	0.6	18.2	1.728
950	2.0	120	0.08	0.9	17.9	1.062
950	2.0	120	0.16	0.3	17.3	1.238
950	2.0	120	0.24	0.6	16.9	1.417

The results obtained for tool life and surface roughness as per Table 4 are subjected to various statistical analysis for further understanding and correlating various factors.

#### 3.2.1. Analysis of tool life.

Table 5 provides the ANOVA results for tool life of annealed steel.

**Table 5:** ANOVA results of Tool Life of Annealed AISI 1040 steel.

Factor	DF	Adj. SS	Adj. MS	F – Value	P – Value	% Contribution
Temperature (°C)	2	58.3622	29.1811	5124.49	0.000	69.21
Time (h)	2	0.0556	0.0278	4.88	0.022	0.07
Speed	2	20.9689	10.4844	1841.17	0.000	24.87
Feed	2	4.8467	2.4233	425.56	0.000	5.75
Depth of Cut	2	0.0022	0.0011	0.20	0.825	0.00
Error	16	0.0911	0.0057	—	—	0.11
Total	26	84.3267	—	—	—	100.00

The ANOVA results for tool life under annealed conditions reveal that temperature, cutting speed, and feed rate are statistically significant factors. Among these, temperature exhibits the highest contribution, followed by cutting speed and feed rate, while depth of cut is found to be insignificant.

Tool life is observed to increase with increase in heat treatment temperature, which can be attributed to the formation of a softer microstructure due to grain coarsening. This reduces cutting resistance and tool wear. In contrast, increasing cutting speed leads to a reduction in tool life due to higher temperature at the cutting interface, which accelerates wear mechanisms such as abrasion and diffusion. Similarly, higher feed rates increase cutting forces, resulting in rapid tool degradation.

The reduction in tool life can be explained by the combined action of mechanical and thermal wear mechanisms. At higher cutting speeds, the temperature at the tool chip interface increases, which accelerates thermal softening of the cutting edge and promotes diffusion-related wear. Higher feed rates increase the cutting load and contact pressure, thereby intensifying abrasive wear and flank wear progression. In annealed specimens, the softer ferrite–pearlite structure offers lower cutting resistance, leading to slower flank wear development. In normalized specimens, the finer ferrite–pearlite structure increases strength and cutting resistance, resulting in moderately higher tool wear. In oil-quenched specimens, the presence of harder martensitic regions increases abrasion and edge loading, thereby reducing tool life significantly. Adhesive wear may also occur due to intermittent material transfer between the workpiece and tool surface, especially under dry cutting conditions.

#### 3.2.2. Analysis of surface roughness.

From the ANOVA results for surface roughness provided in Table 6 indicate that feed rate is the most dominant factor, followed by cutting speed and temperature. The effect of soaking time and depth of cut is statistically insignificant.

**Table 6:** ANOVA results of Surface Roughness of Annealed AISI 1040 steel.

Factor	DF	Adj. SS	Adj. MS	F – Value	P – Value	% Contribution
Temperature (°C)	2	0.16797	0.083984	157.25	0.000	16.14
Time (h)	2	0.00150	0.000749	1.40	0.275	0.14
Speed	2	0.32819	0.164094	307.24	0.000	31.53
Feed	2	0.53455	0.267277	500.43	0.000	51.35
Depth of Cut	2	0.00005	0.000025	0.05	0.954	0.00
Error	16	0.00855	0.000534	—	—	0.82
Total	26	1.04080	—	—	—	100

Surface roughness increases significantly with feed rate due to the formation of deeper feed marks and increased plastic deformation. Lower cutting speeds produce better surface finish due to reduced vibration and thermal effects. The influence of heat treatment temperature on surface roughness is associated with changes in material hardness and deformation characteristics.

### 3.3. Regression analysis for annealed condition

The regression equations is developed to predict the tool life involving various parameters considered for this study with the same range of values.


$$\begin{array}{c} \text{Tool Life }(\text{min})\;=\;-\text{45}.\text{01 }+\;\text{0}.\text{07044}(\text{A})\;+\;\text{0}.\text{056 }(\text{B})\;-\;\text{0}.\text{02694}(\text{ C})\;-\;\text{6}.\text{46 }(\text{D})\;-\;\text{0}.\text{019 }(\text{E}) \end{array}$$ (1)

Equation 1 gives the regression equation where various terms are as follows

A- Temperature in °C, B- Time in h, C- Speed in m/min, D- Feed in mm/rev and E- Depth of Cut in mm.

The regression model developed for tool life shows a high coefficient of determination (R² =96.71%), indicating excellent agreement between predicted and experimental values. The positive coefficient for temperature confirms its beneficial effect on tool life, whereas negative coefficients for cutting speed and feed indicate their adverse influence.

Equation 2 provides the regression equation for surface roughness


$$\begin{array}{cc} & \text{Surface Roughness }=\;-\text{12}.\text{10 }+\;\text{0}.\text{01676}(\text{A})\;+\text{ 17}.\text{49}(\text{B})\;-\;\text{0}.\text{1751}(\text{C})\;+\;\text{7}.\text{7}(\text{D})\;-\;\text{1}.\text{79}(\text{E})\;\\ & -\;\text{0}.\text{02013}(\text{A}\times \text{B})\;+\;\text{0}.\text{000167}(\text{A}\times \text{C})\;-\;\text{0}.\text{0068}(\text{A}\times \text{D})\;+\;\text{0}.\text{00155}(\text{A}\times \text{E})\;+\;\text{0}.\text{01043}(\text{B}\times \text{C})\;\\ & +\;\text{0}.\text{058}(\text{B}\times \text{D})\;+\;\text{0}.\text{041}(\text{B}\times \text{E})\;-\;\text{0}.\text{0005}(\text{C}\times \text{D})\;+\;\text{0}.\text{00053}(\text{C}\times \text{E})\;+\;\text{1}.\text{27}(\text{D}\times \text{E}) \end{array}$$ (2)

Equation (2) gives the regression equation for surface roughness, where the various terms are as follows:

A- Temperature in °C, B- Time in h, C- Speed in m/min, D- Feed in mm/rev and E- Depth of Cut in mm

The regression model for surface roughness includes interaction terms, suggesting that surface finish is influenced by combined effects of machining parameters. Although the model shows good fit (R^2^ = 89.14%), the lower predicted R^2^ indicates some variability due to complex interactions.

The adequacy of the regression models was assessed using the coefficient of determination and the agreement between experimental and predicted responses. The high R^2^ values obtained for tool life and surface roughness indicate that the developed models explain most of the variability in the measured responses. The adjusted R^2^ values were also reasonably close to the corresponding R^2^ values, confirming that the models were not excessively influenced by unnecessary terms. In addition, the residuals were checked to ensure that no abnormal deviation or systematic trend was observed within the experimental domain. However, it should be noted that the present models were developed using the L27 experimental dataset, and independent confirmation experiments outside this design were not performed. Therefore, the regression equations are recommended for prediction within the selected parameter range, while further validation using additional unseen experimental trials may be carried out in future studies.

### 3.4. Optimization of machining parameters

Optimization was carried out using response surface methodology with the objective of maximizing tool life and minimizing surface roughness. The optimal parameters obtained for annealed condition are:

- Temperature: 936°C
- Time: 2 h
- Speed: 112.3 m/min
- Feed: 0.08 mm/rev
- Depth of cut: 0.3 mm

These results indicate that lower feed rate and moderate cutting speed are essential for achieving improved machinability.

### 3.5. Main effects plot interpretation

The main effects plots provide a graphical representation of the influence of input parameters on response variables.

**Fig 1:**
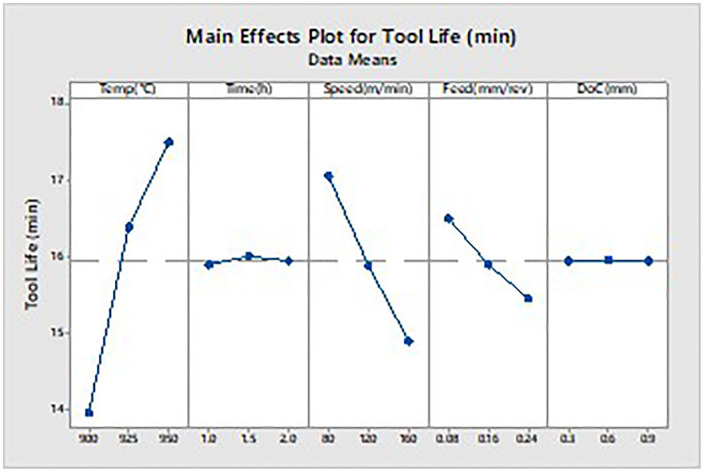
Main effects plot for Tool life of Annealed AISI 1040 steel.

**Fig 2:**
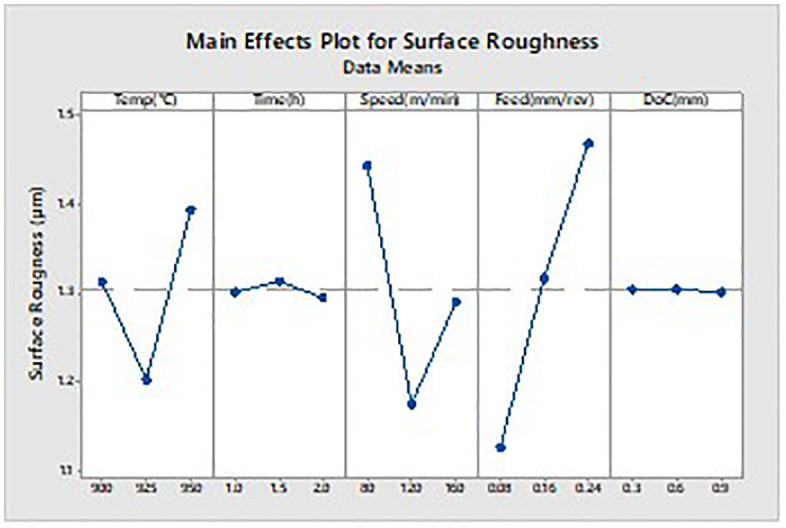
Main effects plot for surface roughness of annealed AISI 1040 steel.

Figs 1 and 2 provide the main effects plot for tool life and surface roughness respectively for annealed conditions.

It may be seen from the Figs 1 and 2 that

- **Tool Life:** Decreases with increasing cutting speed and feed, while it increases with higher heat treatment temperature.
- **Surface Roughness:** Strongly increases with feed rate, indicating its dominant influence on surface quality.

The trends observed confirm the statistical findings and highlight the importance of parameter selection for process optimization.

### 3.6. Hardness results for normalized condition

The Vickers microhardness details with different temperatures and time combinations are provided in the Table 7 below.

**Table 7:** Hardness results for Normalized AISI 1040 steel.

Temperature (°C)	Time (h)	Hardness (HV)
900	1	205
900	1.5	203
900	2	201
925	1	200
925	1.5	197
925	2	195
950	1	195
950	1.5	193
950	2	192

The hardness results presented in Table 7 show that these are slightly higher than that of annealed condition hardness. This may be further verified with the slightly finer grains produced in normalizing compared to annealed conditions because of the moderate cooling method adopted. Further, these results are linked to the various in machinability of the samples.

### 3.7. Machinability results for normalized condition

The results for normalized specimens are provided in Table 8 which show a similar trend to annealed condition but with relatively reduced tool life values.

**Table 8:** Tool Life and Surface Roughness results for normalized AISI 1040 steel.

Temperature (°C)	Time (h)	Speed (m/min)	Feed(mm/rev)	DoC (mm)	Tool Life (min)	Surface Roughness (µm)
900	1.0	80	0.08	0.3	14.1	1.182
900	1.0	80	0.16	0.6	13.6	1.342
900	1.0	80	0.24	0.9	13.2	1.498
900	1.5	120	0.08	0.3	13.3	0.968
900	1.5	120	0.16	0.6	12.7	1.126
900	1.5	120	0.24	0.9	12.2	1.284
900	2.0	160	0.08	0.3	12.3	1.048
900	2.0	160	0.16	0.6	11.8	1.208
900	2.0	160	0.24	0.9	11.3	1.362
925	1.0	120	0.08	0.6	15.2	0.842
925	1.0	120	0.16	0.9	14.6	0.998
925	1.0	120	0.24	0.3	14.2	1.152
925	1.5	160	0.08	0.6	14.3	0.918
925	1.5	160	0.16	0.9	13.7	1.076
925	1.5	160	0.24	0.3	13.2	1.228
925	2.0	80	0.08	0.6	16.4	1.092
925	2.0	80	0.16	0.9	15.8	1.248
925	2.0	80	0.24	0.3	15.4	1.402
950	1.0	160	0.08	0.9	15.3	1.142
950	1.0	160	0.16	0.3	14.7	1.296
950	1.0	160	0.24	0.6	14.3	1.452
950	1.5	80	0.08	0.9	17.2	1.258
950	1.5	80	0.16	0.3	16.6	1.468
950	1.5	80	0.24	0.6	16.2	1.612
950	2.0	120	0.08	0.9	16.0	0.982
950	2.0	120	0.16	0.3	15.4	1.142
950	2.0	120	0.24	0.6	15.0	1.298

The results provided in Table 8 are further analyzed using statistical analysis. ANOVA, regression equations and optimization processes are carried out using Minitab software.

#### 3.7.1. Tool Life Behavior for normalized condition.

ANOVA results from Table 9 indicate that temperature, cutting speed, and feed rate are significant factors, while soaking time and depth of cut remain insignificant. Compared to annealed condition, tool life is lower due to the presence of finer and stronger microstructure.

**Table 9:** ANOVA results of Tool Life of Normalized AISI 1040 steel.

Factor	DF	Adj. SS	Adj. MS	F – Value	P – Value	% Contribution
Temperature (°C)	2	40.1385	20.0693	2016.26	0.000	64.49
Time (h)	2	0.0030	0.0015	0.15	0.863	0.00
Speed	2	17.2985	8.6493	868.95	0.000	27.79
Feed	2	4.6319	2.3159	232.67	0.000	7.44
Depth of Cut	2	0.0052	0.0026	0.26	0.774	0.01
Error	16	0.1593	0.0100	—	—	0.26
Total	26	62.2363	—	—	—	100.00

#### 3.7.2. Surface roughness behavior for normalized condition.

From the Table 10, it is observed that feed rate remains the dominant parameter affecting surface roughness. The normalized structure results in slightly higher roughness compared to annealed condition due to increased material strength.

**Table 10:** ANOVA results of Surface Roughness of Normalized AISI 1040 steel.

Factor	DF	Adj. SS	Adj. MS	F – Value	P – Value	% Contribution
Temperature (°C)	2	0.162848	0.081424	149.04	0.000	17.58
Time (h)	2	0.001495	0.000748	1.37	0.283	0.16
Speed	2	0.299938	0.149969	274.51	0.000	32.37
Feed	2	0.453295	0.226648	414.87	0.000	48.93
Depth of Cut	2	0.000119	0.000060	0.11	0.897	0.01
Error	16	0.008741	0.000546	—	—	0.94
Total	26	0.926438	—	—	—	100.00

#### 3.7.3. Regression and optimization.


$$\begin{array}{c} \text{Tool Life }(\text{min})\;=\;-\text{35}.\text{59 }+\;\text{0}.\text{05822}(\text{A})\;+\;\text{0}.\text{022}(\text{B})\;-\;\text{0}.\text{02444}(\text{C})\;-\;\text{6}.\text{319}(\text{D})\;+\;\text{0}.\text{019}(\text{E}) \end{array}$$ (3)

Equation (3) gives the regression equation for tool life under the normalized condition, where the various terms are as follows:

**A** – Temperature in °C, **B** – Time in h, **C** – Speed in m/min, **D** – Feed in mm/rev, **E** – Depth of Cut in mm

R square = 96.3%

$$\begin{array}{cc} & \text{Surface Roughness }=\;-\text{10}.\text{99 }+\;\text{0}.\text{01532}(\text{A})\;+\text{ 17}.\text{81}(\text{B})\;-\;\text{0}.\text{1825}(\text{C})\;+\;\text{1}.\text{2}(\text{D})\;-\;\text{0}.\text{0037}(\text{E})\;\\ & -\;\text{0}.\text{02048}(\text{A}\times \text{B})\;+\;\text{0}.\text{000175}(\text{A}\times \text{C})\;+\;\text{0}.\text{0010}(\text{A}\times \text{D})\;+\;\text{0}.\text{01077}(\text{B}\times \text{C})\;+\;\text{0}.\text{008}(\text{B}\times \text{D})\;\\ & -\;\text{0}.\text{00120}(\text{C}\times \text{D}) \end{array}$$ (4)

Equation (4) gives the regression equation for surface roughness under the normalized condition, where the various terms are as follows:

**A** – Temperature in °C, **B** – Time in h, **C** – Speed in m/min, **D** – Feed in mm/rev, **E** – Depth of Cut in mm.

R square = 89%

The regression models show high accuracy confirming their predictive capability. The optimized parameters are like annealed condition, with slight variation in depth of cut.

The optimal parameters obtained for normalized condition are:

- Temperature: 935°C

- Time: 2 h

- Speed: 111.8 m/min

- Feed: 0.08 mm/rev

- Depth of cut: 0.6 mm

### 3.8. Main effects plot interpretation for normalized condition

**Fig 3:**
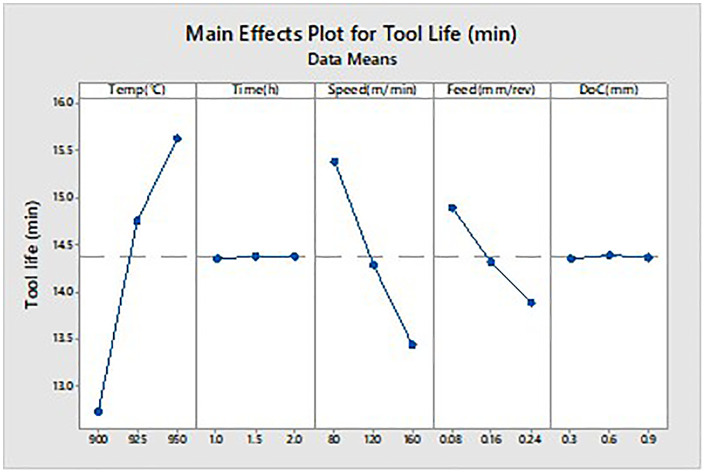
Main effects plot for Tool life of Normalized AISI 1040 steel.

Fig 3 presents the main effects plot for tool life of normalized AISI 1040 steel. The plot clearly shows that temperature has a strong positive influence on tool life. As the normalizing temperature increases from 900°C to 950°C, tool life increases sharply, after which the increase becomes more moderate up to 950°C. This indicates that higher normalizing temperature improves the machinability of the steel under the selected cutting conditions. In contrast, cutting speed has a strong negative effect on tool life, with tool life decreasing continuously as speed increases from 80 to 160 m/min. This behavior is expected because higher cutting speed increases frictional heat and tool wear rate. Feed also shows a negative influence, as tool life decreases steadily with increase in feed from 0.08 to 0.24 mm/rev, which may be due to the higher cutting load imposed on the tool. On the other hand, holding time and depth of cut exhibit nearly horizontal lines, indicating that their effects on tool life are negligible within the chosen experimental range.

**Fig 4:**
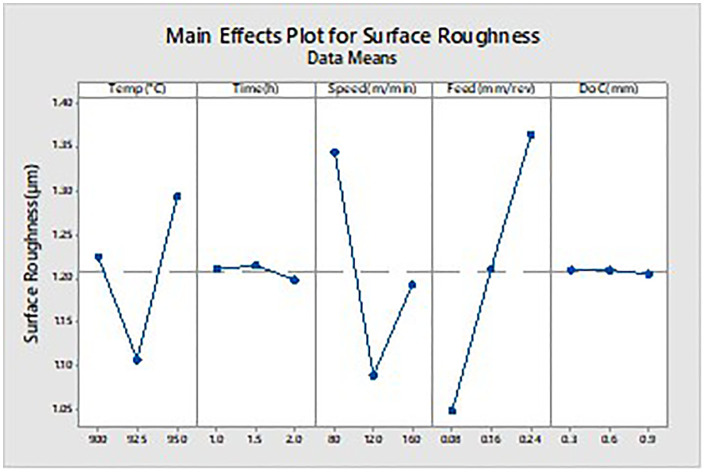
Main effects plot for Surface Roughness of Normalized AISI 1040 steel.

Fig 4 shows the main effects plot for surface roughness of normalized AISI 1040 steel. It is evident that feed has the most dominant effect on surface roughness. As feed increases from 0.08 to 0.24 mm/rev, surface roughness rises sharply, indicating deterioration in surface finish at higher feed values due to the formation of deeper feed marks on the machined surface. Cutting speed also has a significant influence. Surface roughness decreases markedly when speed increases from 80 to 120 m/min, suggesting improved surface finish at intermediate cutting speed, but then increases again at 160 m/min. This indicates that the best surface finish is obtained around the middle speed level. Temperature also affects surface roughness, showing a non-linear trend: roughness decreases from 900°C to 925°C and then increases again at 950°C, indicating that the intermediate temperature condition gives the smoothest surface. In comparison, holding time and depth of cut show almost flat trends, confirming that their effects are very small.

These trends are consistent with the ANOVA results, where temperature, speed, and feed were found to significantly affect tool life, while feed and speed were the most influential factors for surface roughness.

### 3.9. Hardness results for oil quenched condition

The Vickers microhardness details with different temperatures and time combinations are provided in Table 11 below

**Table 11:** Hardness results for Oil quenched AISI 1040 steel.

Temperature (°C)	Time (h)	Hardness (HV)
900	1	245
900	1.5	240
900	2	239
925	1	230
925	1.5	220
925	2	215
950	1	210
950	1.5	200
950	2	196

The higher hardness obtained as shown in Table 11 is attributed to the formation of martensite because of the rapid quenching in the oil.

### 3.10. Machinability results for oil quenched condition

The results for oil quenched specimens are provided in Table 12 which indicate a significant reduction in machinability compared to annealed and normalized conditions.

**Table 12:** Tool Life and Surface Roughness results for oil quenched AISI 1040 steel.

Temperature (°C)	Time (h)	Speed (m/min)	Feed(mm/rev)	DoC (mm)	Tool Life (min)	Surface Roughness (µm)
900	1.0	80	0.08	0.3	12.6	1.128
900	1.0	80	0.16	0.6	12.1	1.276
900	1.0	80	0.24	0.9	11.7	1.432
900	1.5	120	0.08	0.3	11.8	0.926
900	1.5	120	0.16	0.6	11.2	1.078
900	1.5	120	0.24	0.9	10.7	1.236
900	2.0	160	0.08	0.3	10.9	1.006
900	2.0	160	0.16	0.6	10.4	1.162
900	2.0	160	0.24	0.9	9.9	1.316
925	1.0	120	0.08	0.6	13.6	0.812
925	1.0	120	0.16	0.9	13.0	0.964
925	1.0	120	0.24	0.3	12.6	1.118
925	1.5	160	0.08	0.6	12.7	0.886
925	1.5	160	0.16	0.9	12.1	1.038
925	1.5	160	0.24	0.3	11.6	1.188
925	2.0	80	0.08	0.6	14.5	1.058
925	2.0	80	0.16	0.9	13.9	1.214
925	2.0	80	0.24	0.3	13.5	1.368
950	1.0	160	0.08	0.9	13.6	1.108
950	1.0	160	0.16	0.3	13.0	1.258
950	1.0	160	0.24	0.6	12.6	1.412
950	1.5	80	0.08	0.9	15.2	1.226
950	1.5	80	0.16	0.3	14.6	1.428
950	1.5	80	0.24	0.6	14.2	1.572
950	2.0	120	0.08	0.9	14.1	0.942
950	2.0	120	0.16	0.3	13.5	1.098

#### 3.10.1. Tool life behavior for oil quenched condition.

From the results provided in Table 13, it is noticed that tool life is considerably lower in quenched samples due to the presence of hard martensitic structure. ANOVA results confirm that temperature, cutting speed, and feed rate significantly influence tool life. The effect of soaking time is also slightly more pronounced compared to other conditions.

**Table 13:** ANOVA results of Tool Life of Oil Quenched AISI 1040 steel.

Factor	DF	Adj. SS	Adj. MS	F – Value	P – Value	% Contribution
Temperature (°C)	2	30.1541	15.0770	2101.06	0.000	62.33
Time (h)	2	0.0585	0.0293	4.08	0.037	0.12
Speed	2	13.4141	6.7070	934.66	0.000	27.73
Feed	2	4.6319	2.3159	322.74	0.000	9.57
Depth of Cut	2	0.0052	0.0026	0.36	0.702	0.01
Error	16	0.1148	0.0072	—	—	0.24
Total	26	48.3785	—	—	—	100.00

#### 3.10.2. Surface roughness behavior oil quenched condition.

From the results provide in ANOVA Table 14, it may be seen that surface roughness values are relatively higher due to increased cutting forces and unstable cutting conditions. Feed rate remains the most influential parameter.

**Table 14:** ANOVA results of Surface Roughness of Oil Quenched AISI 1040 steel.

Factor	DF	Adj. SS	Adj. MS	F – Value	P – Value	% Contribution
Temperature (°C)	2	0.151837	0.075918	96.10	0.000	17.01
Time (h)	2	0.001467	0.000733	0.93	0.415	0.16
Speed	2	0.290462	0.145231	183.84	0.000	32.54
Feed	2	0.436217	0.218109	276.09	0.000	48.86
Depth of Cut	2	0.000107	0.000053	0.07	0.935	0.01
Error	16	0.012640	0.000790	—	—	1.42
Total	26	0.892729	—	—	—	100.00

#### 3.10.3. Regression and optimization.


$$\begin{array}{c} \text{Tool Life }(\text{min})\;=\;-\text{30}.\text{01 }+\;\text{0}.\text{05022}(\text{A})\;-\;\text{0}.\text{111}(\text{B})\;-\;\text{0}.\text{02153}(\text{C})\;-\;\text{6}.\text{319}(\text{D})\;+\;\text{0}.\text{019}(\text{E}) \end{array}$$ (5)

Equation (5) gives the regression equation for tool life under the oil quenched condition, where the various terms are as follows:

**A** – Temperature in °C, **B** – Time in h, **C** – Speed in m/min, **D** – Feed in mm/rev, **E** – Depth of Cut in mm

R square= 95.8%

$$\begin{array}{cc} & \text{Surface Roughness }=\;-\text{12}.\text{71 }+\;\text{0}.\text{01705}(\text{A})\;+\text{ 16}.\text{77}(\text{B})\;-\;\text{0}.\text{1574}(\text{C})\;+\text{ 13}.\text{0}(\text{D})\;-\;\text{3}.\text{40}(\text{E})\;\\ & -\;\text{0}.\text{01916}(\text{A}\times \text{B})\;+\;\text{0}.\text{000150}(\text{A}\times \text{C})\;-\;\text{0}.\text{0128}(\text{A}\times \text{D})\;+\;\text{0}.\text{00321}(\text{A}\times \text{E})\;+\;\text{0}.\text{00914}(\text{B}\times \text{C})\;\\ & -\;\text{0}.\text{042}(\text{B}\times \text{D})\;+\;\text{0}.\text{016}(\text{B}\times \text{E})\;-\;\text{0}.\text{00153}(\text{C}\times \text{D})\;+\;\text{0}.\text{00050}(\text{C}\times \text{E})\;+\;\text{1}.\text{87}(\text{D}\times \text{E}) \end{array}$$ (6)

Equation (6) gives the regression equation for surface roughness under the oil-quenched condition, where the various terms are as follows:

**A** – Temperature in °C, **B** – Time in h, **C** – Speed in m/min, **D** – Feed in mm/rev, **E** – Depth of Cut in mm

R square= 90%

The regression models show excellent fit indicating strong predictive capability. The optimized parameters indicate the need for low feed and moderate cutting speed even in harder materials.

The optimized parameters are also in a similar range of normalized and annealed conditions.

### 3.11. Main effects plot interpretation for oil quenched condition

**Fig 5:**
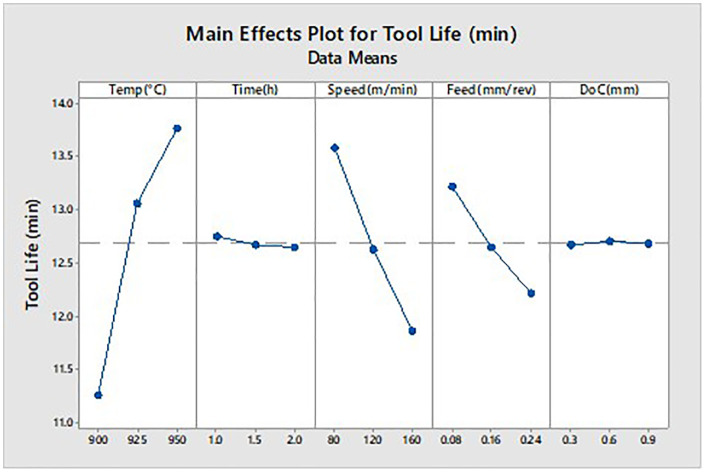
Main effects plot for Tool Life of Oil Quenched AISI 1040steel.

**Fig 6:**
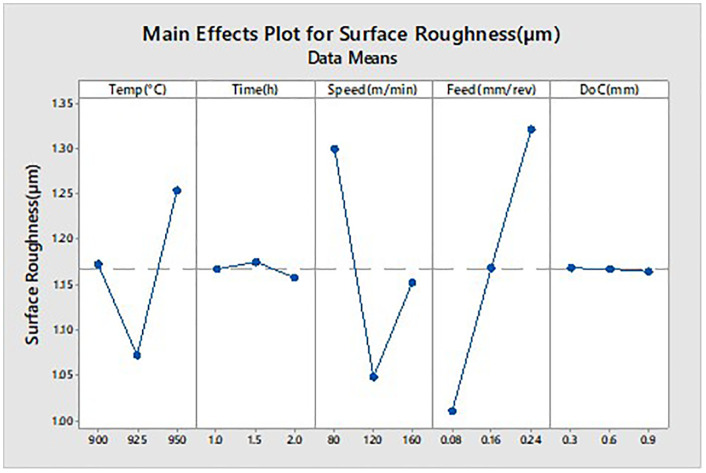
Main effects plot for Surface Roughness of Oil Quenched AISI 1040steel.

Figs 5 and 6 provide the main effects plot for tool life and surface roughness for oil quenched conditions respectively. It may be seen that the trend agrees with the ANOVA results.

### 3.12. Microstructural analysis (SEM)

The SEM observations clearly support the experimental results and confirm that microstructure plays a critical role in determining machinability.

**Fig 7:**
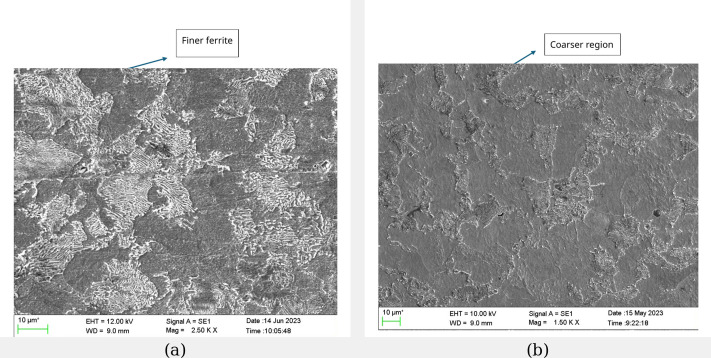
SEM images of Annealed AISI 1040 steel at a temperature of a) 900°C b) 950°C.

Fig 7 shows the SEM micrographs of annealed AISI 1040 steel at 900°C and 950°C. The annealed specimens exhibit a typical ferrite–pearlite microstructure, which is characteristic of slow furnace cooling. In Fig 7(a), corresponding to 900°C, the microstructure appears relatively finer, with distinguishable ferrite regions and pearlitic colonies. In Fig 7(b), at 950°C, the microstructure becomes comparatively coarser due to enhanced grain growth during heating and the slow cooling associated with annealing. The presence of coarse ferrite–pearlite structure reduces hardness and cutting resistance, which explains the superior machinability observed for annealed samples. This softer microstructure promotes lower tool wear and improved tool life, making the annealed condition the most favorable among the three heat treated states.

**Fig 8:**
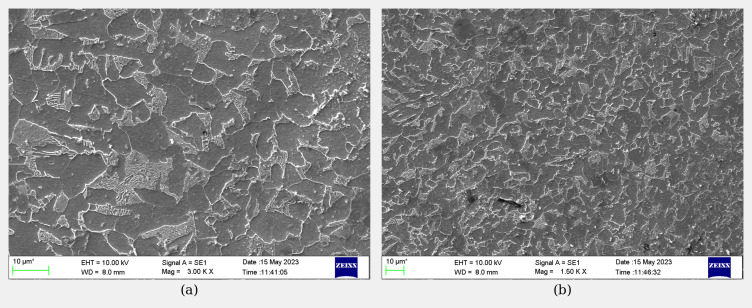
SEM images of Normalized AISI 1040 steel at a temperature of a) 900°C b) 950°C.

Fig 8 presents the SEM micrographs of normalized AISI 1040 steel at 900°C and 950°C. Compared with the annealed condition, the normalized specimens show a finer and more uniform ferrite–pearlite structure due to air cooling from the austenitizing temperature. The refinement of grains is more evident than in the annealed samples, indicating that the cooling rate in normalizing suppresses excessive grain coarsening and produces a relatively homogeneous microstructure. Such a refined structure results in slightly higher hardness than the annealed state and therefore offers intermediate machinability. As a result, normalized specimens show lower tool life than annealed samples but better performance than oil quenched specimens. Thus, the SEM observations of Fig 8 support the experimental findings that normalizing provides a balanced combination of strength and machinability.

**Fig 9:**
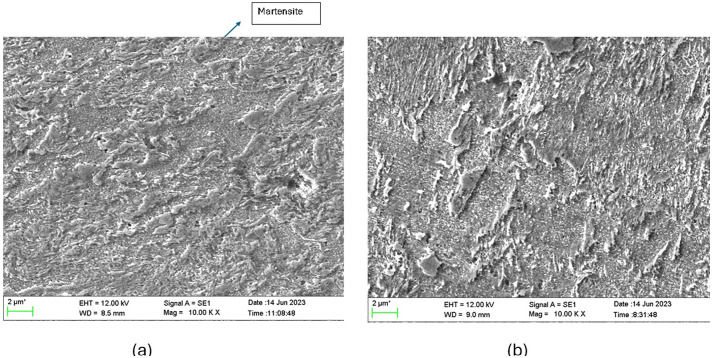
SEM images of Oil quenched AISI 1040 steel at a temperature of a) 900°C b) 950°C.

Fig 9 shows the SEM micrographs of oil quenched AISI 1040 steel at 900°C and 950°C. The quenched specimens reveal the formation of martensitic structure, produced by rapid cooling in SAE 40 oil. This microstructure is much harder and more brittle than the ferrite–pearlite structures observed in annealed and normalized samples. The martensitic regions visible in the SEM image indicate a transformation-dominated microstructure with high hardness, which directly increases cutting resistance during machining. Consequently, the oil quenched condition exhibits the lowest tool life and relatively poor machinability among the three conditions. The harder martensitic phase also contributes to increased tool wear and unstable cutting action, thereby adversely affecting surface finish. Therefore, the SEM features in Fig 9 clearly explain why the oil-quenched samples produced the least favorable machining performance.

Overall, Figs 7 to 9 confirm that the progressive change in microstructure from coarse ferrite–pearlite in the annealed condition, to refined ferrite–pearlite in the normalized condition, and finally to hard martensite in the oil quenched condition governs the observed variation in hardness and machinability. The annealed condition provides the best machinability due to its softer microstructure, the normalized condition shows moderate behavior because of grain refinement, and the oil quenched condition exhibits poor machinability owing to the formation of martensite and the associated increase in hardness.

The SEM observations provide qualitative evidence for the microstructural changes responsible for the observed machinability trends. The annealed specimens show a relatively softer ferrite–pearlite structure, which is consistent with lower hardness values and improved tool life. The normalized specimens exhibit a finer ferrite–pearlite structure, resulting in slightly higher hardness and intermediate machinability. In contrast, the oil-quenched specimens show martensitic features, which correspond to higher hardness, increased cutting resistance, and reduced tool life. Thus, the hardness results support the qualitative SEM-based interpretation.

From an industrial perspective, the present results are useful for selecting suitable heat treatment and machining conditions for AISI 1040 steel components where both tool economy and surface quality are important. The results indicate that annealed specimens provide better machinability and longer tool life, which can reduce tool replacement frequency and machining cost. However, normalized and oil-quenched conditions may be preferred when higher strength or hardness is required in service, although they involve a compromise in tool life. The optimization results show that lower feed rate and moderate cutting speed are beneficial for achieving improved surface finish and acceptable tool life. In production environments, such parameter selection can reduce excessive tool wear, minimize rework due to poor surface finish, and improve process reliability. Therefore, the findings provide practical guidance for balancing productivity, tool cost, and surface integrity during dry turning of heat-treated AISI 1040 steel.

## 4. Conclusions

A comprehensive investigation was carried out to evaluate the effect of heat treatment and machining parameters on the machinability of AISI 1040 steel under annealed, normalized, and oil-quenched conditions. Based on the experimental results, statistical analysis, regression modeling, optimization, and SEM observations, the following conclusions can be drawn:

1. Heat treatment significantly affected the machinability of AISI 1040 steel through microstructural modification. Among the investigated conditions, the annealed specimens exhibited the best machinability, whereas the oil-quenched specimens showed the poorest machinability because of their higher hardness and martensitic structure.
2. Temperature was the most influential heat-treatment parameter governing tool life in all three conditions. Higher austenitizing temperature improved tool life, while cutting speed and feed rate reduced tool life because of increased cutting temperature, friction, and tool wear.
3. Feed rate was the most dominant factor affecting surface roughness in all heat-treated conditions, followed by cutting speed and temperature. An increase in feed rate caused a marked deterioration in surface finish because of deeper feed marks and greater plastic deformation on the machined surface.
4. Depth of cut showed a negligible influence on both tool life and surface roughness within the selected experimental range, whereas soaking time showed either limited or condition-dependent influence compared with temperature, speed, and feed rate.
5. The developed regression models showed good predictive capability for estimating tool life and surface roughness within the investigated process window, indicating their usefulness for process prediction and parameter selection.
6. SEM analysis established a clear relationship between microstructure and machinability. The coarse ferrite-pearlite structure in the annealed condition favored improved machinability, the refined ferrite-pearlite structure in the normalized condition produced intermediate performance, and the martensitic structure in the oil-quenched condition led to reduced tool life and poorer machining performance.
7. The optimization results indicated that improved machinability is achieved at lower feed rate and moderate cutting speed, while the favorable heat-treatment setting lies toward the higher temperature range considered in the present study.
8. Overall, the study demonstrates that a combined consideration of heat-treatment route and machining parameters is essential for achieving an appropriate balance between productivity, tool performance, and surface quality during machining of AISI 1040 steel.
